# Desmoplastic reaction in the microenvironment of head and neck and other solid tumors: the therapeutic barrier

**DOI:** 10.1177/17588359251317144

**Published:** 2025-02-07

**Authors:** Kohei Okuyama, Maiko Tsuchiya, Kala Chand Debnath, Shajedul Islam, Souichi Yanamoto

**Affiliations:** Department of Head and Neck Surgery, The University of Texas MD Anderson Cancer Center, 1515 Holcombe Blvd., Unit 123, Houston, TX 77030-4009, USA; Department of Pathology, Teikyo University School of Medicine, Itabashi-ku, Tokyo, Japan; Department of Head and Neck Surgery, The University of Texas MD Anderson Cancer Center, Houston, TX, USA; Department of Immunology, The University of Texas MD Anderson Cancer Center, Houston, TX, USA; Department of Oral Oncology, Graduate School of Biomedical and Health Sciences, Hiroshima University, Hiroshima-shi, Hiroshima, Japan

**Keywords:** cancer-associated fibroblasts, desmoplastic reaction, extracellular matrix, head and neck cancer, immune-checkpoint inhibitor, tumor microenvironment

## Abstract

Head and neck squamous cell carcinoma (HNSCC) remains a challenge due to limited prognostic biomarkers and therapeutic options. The tumor microenvironment (TME), particularly the desmoplastic reaction (DR) characterized by stromal fibrosis, plays a crucial role in cancer progression and resistance to therapy. This review aims to summarize the biological significance of DR in HNSCC initiation, progression, and treatment resistance. Histologically, DR in HNSCC correlates with invasion patterns and clinical outcomes, affecting disease-free and overall survival. The interaction between cancer-associated fibroblasts (CAFs) and TME influences immune responses, including resistance to immunotherapy. Notably, human papillomavirus-driven HNSCC exhibits distinct DR characteristics that further influence the prognosis. DR promotes epithelial-mesenchymal transition and cancer cell invasion through CAF-mediated extracellular matrix remodeling and signaling pathways such as transforming growth factor-beta. DR also affects bone invasion and chemotherapy resistance by modulating stromal responses. Therapeutic strategies targeting DR and stromal components show promise in overcoming therapeutic resistance including resistance to immune checkpoint inhibitors. Understanding the role of DR in HNSCC biology and its impact on treatment response is critical to developing effective therapeutic interventions.

## Introduction

Currently, there are limited reliable clinical biomarkers available for stratifying head and neck squamous cell carcinoma (HNSCC) patients according to their prognosis.^1^ In solid malignant tumors, stromal and immune cells constitute fundamental components of the tumor microenvironment (TME), and understanding the status of the TME and intratumoral heterogeneity is a major challenge in oncology. The TME has an important influence on the biological behavior of cancer and the effect of anticancer treatments, and tumor-host interactions are essential for the invasion and metastasis of various cancers. The tumor stroma consists of non-malignant cells, including normal fibroblasts and cancer-associated fibroblasts (CAFs), innate and adaptive immune cells, microvessels, and extracellular matrix (ECM).^2^ The tumor stroma is formed by a complex process induced by tumor-host interactions, and the histological features of the tumor stroma may serve as independent prognostic factors in various solid tumors.^3^

Desmoplastic reaction (DR) is defined as the growth of fibrous or connective tissues at sites of stromal invasion by cancer,^4^ consisting of fibroblasts, lymphatic and vascular endothelial cells, immune cells, pathologically increased nerves, and ECM, forming a complex TME that promotes cancer development, invasion, metastasis, and resistance to anticancer therapies. DR refers to the growth of excessive stromal tissue around tumors, and CAFs are key players in the cancer stroma and are a typical sign of aggressiveness in various types of cancers, including HNSCCs.^5–11,12^

DR in the TME may also provide a negative indication for immune checkpoint inhibition that rely on endogenous T cells have proven challenging for the treatment of many types of solid tumors.^13,14^ The key components of the immunosuppressive TME in solid tumors include CAFs and associated DR tissues.^15–17^ This review aims to summarize the current evidence on the role of DR in cancer, focusing on its biological role and function in HNSCC initiation, progression, and treatment resistance and emphasizing the therapies for targeting tumor stroma.

## Clinicopathological features for DR

**Figure 1.**
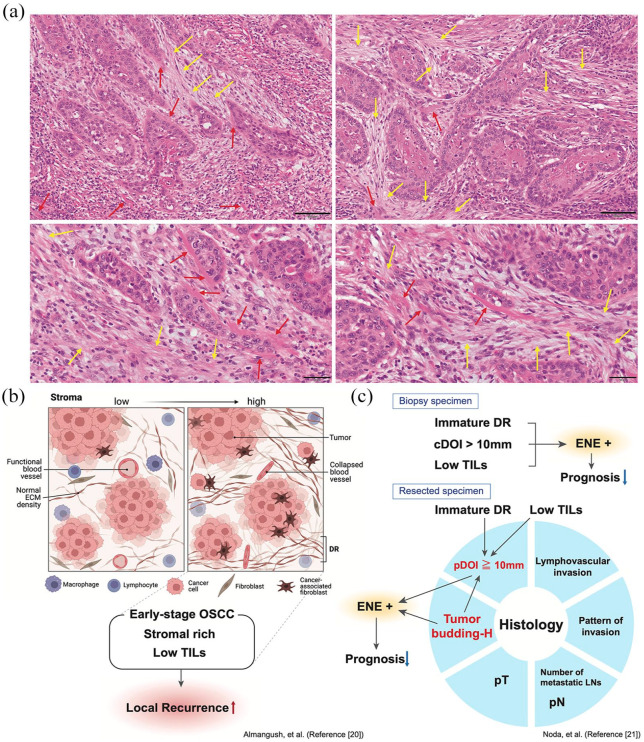
(a) Representative microscopic H&E images of DR in human HNSCC samples (Tongue SCC, pT4). The yellow arrows show myxoid stroma, which is relatively typical of an immature type, including fibroblasts (shown as nuclei-positive cells). The red arrows show the collagenic changes in the DR. The lower images show higher magnification. Tumor nests are surrounded by collagenic stromal tissues, indicating obvious evidence between tumor growth and stromal reactions. ×200, scale bar, 100 µm (upper panel); ×400, scale bar, 50 µm (lower panel). (b) The representative schema of stroma-low and high TME. The stromal-rich and low TIL in early-staged oral SCC is the significant factor for local recurrence. (c) Clinicopathological factors for DR formation and its relation to ENE occurrence in oral SCC. Immature DR, cDOI >10 mm, and low TILs in the biopsy specimen are significant factors for ENE. The high tendency of tumor budding and pathologically diagnosed DOI >10 mm in resection specimens are potent predictors of ENE, that directly affect the prognosis of the disease. cDOI, clinically diagnosed depth of invasion; DR, desmoplastic reaction; ENE, extranodal extension; HNSCC, head and neck squamous cell carcinoma; SCC, squamous cell carcinoma; TIL, tumor-infiltrating lymphocyte; TME, tumor microenvironment.

Histopathologically, DR is characterized by the presence of a fibrotic or myxoid stroma induced by tumor invasion. Ueno et al.^7^ classified DR into three patterns: mature, absence of keloid-like collagen or myxoid stroma; intermediate, presence of keloid-like collagen; and immature, presence of myxoid change (Figure 1(a)). In colorectal cancer (CRC), Shin et al. reported no difference in the 5-year survival of patients with different desmoplastic maturation status. However, they confirmed a difference in the mechanism of tumor invasion: when mature DR was observed, more lymphatic invasion occurred. Conversely, immature fibrosis of the tumor stroma promotes tumor growth. These various cancer invasion routes may be due to the mature or immature stroma, which may also dilute the prognostic effect of desmoplastic maturation. Their study concluded that the maturity of CAFs and DR were associated with tumor-infiltrating growth patterns and cancer invasions, such as lymphatic invasion and tumor budding.^18^

To date, the research has been limited on DR in HNSCC compared to that in other solid tumors, possibly because the total number of cases is lower compared to cancers of other organs like lung, gastric, colorectal, and pancreatic ductal adenocarcinoma (PDAC). Recently, Amano et al. suggested a novel histological classification of oral SCC stroma patterns into groups based on the presence or absence of DR, a diffuse immune/inflammatory reaction, keloid-like collagen, and/or myxoid stroma at the invasive front of the tumor and revealed that these patterns were significantly associated with various clinicopathological factors, such as the histological grade, lymphovascular invasion, perineural invasion, and a diffuse invasion pattern. They also found that this category was identified as an independent prognostic factor for recurrence-free survival and overall survival (OS).^19^ Almangush et al. previously categorized the tumor stroma based on the degree of DR (immature, intermediate, or mature) in early-stage oral SCC. This revealed that a high amount of stroma with a weak infiltration of lymphocytes was significantly associated with worse disease-free survival with a hazard ratio (HR) of 2.68 (95% CI 1.26–5.69), worse OS (HR 2.95, 95% CI 1.69–5.15), and poor disease-specific survival (HR 2.66, 95% CI 1.11–6.33). Such tumors were also significantly associated with a high rate of local recurrence (HR 4.13, 95% CI 1.67–10.24), but no significant association was found with lymph node metastasis (HR 1.27, 95% CI 0.37–4.35). On the other hand, they also found that the categorization of the stroma based on such DR status showed a low prognostic value for early oral tongue SCC in all survival analyses (Figure 1(b)).^20^ Moreover, Noda et al. demonstrated a histopathological association between biopsy and resected oral SCC samples. They found that the TME status in primary tumors was significantly associated with extranodal extension (ENE), and high numbers of tumor budding (TB-H, 10 > TB number in a ×20 objective) were independent risk factors for ENE. Moreover, the histological status of immature DR/low TILs/clinically diagnosed depth of invasion (DOI) > 10 mm in biopsy specimens and TB-H, as well as pathologically diagnosed DOI > 10 mm in resection specimens, are potent predictors of ENE, which directly affect the prognosis of the disease. The statistical analysis of their cohort revealed that pDOI ⩾10 mm, metastasis, number of lymph nodes, pattern of invasion, lymphovascular invasion, pT, and pN status were significantly associated with DR formation in the primary tumor (Figure 1(c)).^21^ Zainab et al. investigated the correlation between the stages of oral SCC and DR status. They concluded that in the initial stages, cancer invasion induces DR, which involves the formation of new ECM through the activation of stromal cells. In turn, in advanced stages, the mechanical pressure exerted by tumor growth and proteolysis induced by the tumor results in collagen degradation, which facilitates deeper invasion of the tumor.^22^ However, this conclusion has limitations; it might be accurate when looking at changes in a fixed tumor area. Since the leading edge of tumor invasion keeps expanding outward as the tumor grows, DR can be formed differently as the invasion front changes.

In CRC, DR characterization was identified as an independent prognostic factor, along with T stage and tumor budding. Determining whether DR is mature or immature is the single best discriminator of recurrence-free survival events and DR type is the only factor that can define one prognostic subgroup by itself.^23^ Resultingly, stromal factors are essential for prognostic determination, consistent with recent molecular biology studies indicating that genes associated with poor prognosis are expressed by stromal cells rather than epithelial cancer cells.^23^

In addition, recent developments in diagnostic strategies are also noteworthy. Vidiri et al. investigated DR detection methods using kurtosis imaging (DKI) and dynamic contrast-enhanced (DCE)-MRI in patients with oral SCC. They found significant differences in DCE-MRI and DKI parameters related to inflammatory infiltrate, tumor grading, keratinization, and DR.^24^ This imaging strategy can be employed to detect DR preoperatively, potentially aiding in the decision-making process for adjuvant therapy.

Despite the abovementioned clear evidence, DR is rarely considered in current clinical guidelines and adjuvant chemotherapy decisions for various types of solid cancers. The future addition of DR evaluation to pathology, additional inclusion of treatment guidelines, and the development of therapeutic approaches for DR are expected.

## Human papillomavirus-driven HNSCC and DR formation

It is well known that the biological characteristics of human papillomavirus (HPV)-related HNSCC differ from those of HPV-negative HNSCC. Several studies have investigated, from a pathological perspective, how the HPV status in HNSCC influences the formation of DR.

Channir et al.^25^ reported that, despite the similar clinical features of HPV-driven and HPV-negative oropharyngeal SCC, HPV-driven oropharyngeal SCC that invades the lymphoid compartment induces stromal DR and tumor infiltration of the peritonsillar striated muscle, and associated DR has also been detected. They also classified the invasion pattern at the leading edge of the tumor as cohesive or non-cohesive, revealing that tumors with a cohesive invasive pattern were more common in HPV+ diseases (60% (HPV+) vs 40% (HPV−)), in contrast to the non-cohesive invasive pattern being more common in HPV− diseases (43% (HPV+) vs 57% (HPV−)). Regarding neck lymph node metastases, they reported that 94% of the metastases in their cohort were p16-positive, HPV DNA-positive, and HPV mRNA ISH-positive, which are also characterized as non-keratinizing SCC. This evidence underscores the role of non-keratinizing SCC in neck lymph node metastases, which most likely indicates an occult HPV-driven primary HNSCC.^25^ At the same time, this observation further highlights the close association between DR status and tumor keratinization.

Kürten et al. conducted a single-cell RNA sequencing (scRNA-seq) to explore the heterogeneity, unique signatures, and cell-cell interactions among immune and non-immune cell populations in HPV+ and HPV− HNSCC tumors and matched peripheral blood samples. Their findings revealed that fibroblasts with elastic differentiation in the HPV+ TME are associated with poor prognosis. Additionally, they identified therapeutically targetable checkpoint receptor-ligand interactions and demonstrated that tumor-associated macrophages are major contributors to programmed death ligand 1 (PD-L1) expression and other immune checkpoint ligands within the HNSCC TME.^26^

More detailed molecular and biological evaluations of the p16-mediated intracellular processes, such as ubiquitination, and its extracellular effects, including microRNA secretion, in HPV+ cancer with DR are needed.

## CAFs and DR contributions to the tumor growth and epithelial-mesenchymal transition

One factor that influences the prognosis of DR is its involvement in the epithelial-mesenchymal transition (EMT): DR is associated with EMT in cancer cells.^27^ In CRC, Shiraishi et al.^28^ reported that smaller-sized pT4 tumors were paradoxically associated with non-mature DR and with EMT-related histology, which means that a tumor that has acquired an invasive phenotype at an early stage and is characterized by a smaller tumor size may be prone to recurrence or metastasis and lead to poor survival. Moreover, keloid-like collagen and myxoid stroma are rarely observed at the center of tumors but appear exclusively at the invasive front of tumors, where remodeling of the TME occurs, and EMT-like histology is often observed in highly aggressive tumors.^23^ There is also a close link between DR and the degree of tumor budding, a process closely associated with EMT-related gene expression.^7,29–31^

CAFs derived either from resident fibroblasts or tumor-infiltrating mesenchymal stem cells are a major component of the stroma in cancers with DR. Moreover, DR may represent the histological consequences of ECM remodeling generated by CAFs.^4^

In the TME, CAFs also induce EMT in neoplastic cells, thus favoring invasiveness.^12,32^ Transforming growth factor-beta (TGF-β) is a well-established driver of EMT,^33,34^ and the relationship between TGF-β and CAFs has also been well-investigated. Evidence shows a relationship between stromal collagen status and TGF-β signaling. Vázquez-Villa et al. found that *COL11A1*/(pro)collagen 11A1 is highly expressed in activated stromal cells of the DR of different human invasive carcinomas, and this expression is correlated with cancer aggressiveness, progression, and lymph node metastasis, while COL11A1/(pro)collagen 11A1 is not expressed in stromal cells under normal conditions. *COL11A1* upregulation is associated with TGF-β1, Wnt, and Hh signaling pathways, which are especially active in cancer-associated stromal cells, including CAFs. Thus, *COL11A1*/(pro)collagen 11A1 expression is a remarkable biomarker of human cancer-associated stromal cells, cancer progression, and EMT.^35^ Furthermore, this could be considered an indirect biomarker for DR formation, and this mechanism of action has also been confirmed in HNSCC cases.^36–38^

CAF activation through pathways independent of TGF-β has also been widely reported. In non-melanoma skin cancer, cancer cell-secreted activin A induces a tumor-promoting phenotype in the fibroblast compartment, with distinct properties compared to TGF-β-activated fibroblasts. Moreover, activin A reprograms fibroblasts through the transcriptional regulation of mDia2 and reduction of nuclear p53, which favors CAF marker expression and increases tumor growth and migration.^39^

Recent data have also suggested that CAFs in DR are phenotypically, functionally, and genetically heterogeneous, dynamically influenced by their TME and cellular origins. Costea et al. identified two CAF subtypes in HNSCC with differing tumor-promoting capabilities: CAF-N, which exhibited traits resembling normal fibroblasts, and CAF-D, which showed a divergent expression profile. CAF-N invasion depended on hyaluronan scaffolds or chain elongation, whereas CAF-D produced higher TGF-β1 levels, driving EMT and enhancing HNSCC cell invasion.^40^

Obradovic et al. utilized scRNA-seq on pre- and post-treatment HNSCC patient tumors, identifying 14 distinct cell types with fibroblasts showing significant post-treatment changes after nivolumab therapy. Among fibroblast subtypes, HNCAF-0/3 emerged as predictive of nivolumab response, while HNCAF-1 was linked to immunosuppression. HNCAF-0/3 reduced TGF-β-driven CD8+ T-cell exhaustion, promoted CD103+NKG2A+ resident memory phenotypes, and enhanced cytotoxic T-cell profiles. Their findings highlight the functional significance of HNCAF subsets in modulating the immunoregulatory TME and their potential as biomarkers for predicting therapeutic outcomes in future clinical trials.^41^

Furthermore, Li et al. performed spatial transcriptomic analysis of HNSCC specimens with differing immune infiltration and scRNA-seq of five pairs of tumor and adjacent tissues, revealing specific CAF subsets related to CD8+ T-cell infiltration restriction and dysfunction, revealed these CAFs exhibited high expression of CXCLs (CXCL9, CXCL10, and CXCL12) and MHC-I and galectin-9 (Gal9), and the MHC-IhiGal9+ CAFs population was inversely correlated with abundance of a TCF1+GZMK+ subset of CD8+ T cells. Moreover, the enrichment of Gal9 on CAFs induced CD8+ T-cell dysfunction and decreased the proportion of tumor-infiltrating TCF1+CD8+ T cells. Taken together, they concluded the identification of MHC-IhiGal9+ CAFs advances the understanding of the precise role of CAFs in cancer immune evasion and paves the way for more effective immune checkpoint inhibitors (ICI) therapies for HNSCC.^42^

**Figure 2.**
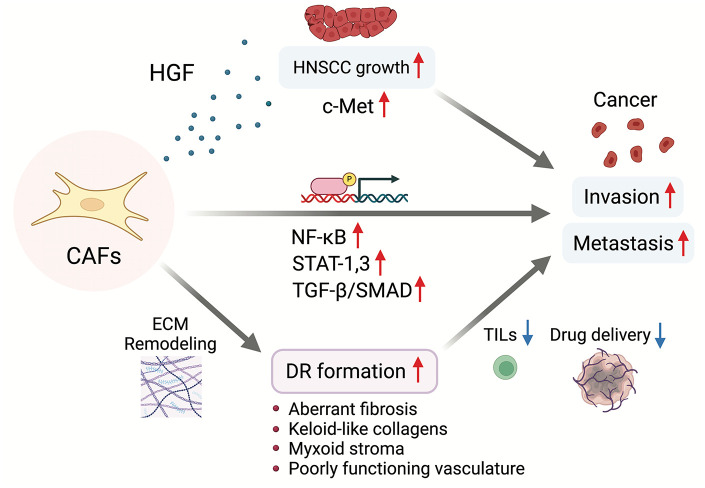
The function and relationship among CAFs, cancer cells, and DR formation in the TME. CAF, cancer-associated fibroblasts; DR, desmoplastic reaction; TME, tumor microenvironment.

CAFs that are categorized into unfavorable DR categories effectively aid tumors in developing a TME beneficial to themselves; aberrant fibrotic components, keloid-like collagens, and myxoid stroma, which are manifestations of CAFs in aggressive tumors, have been reported—namely, it’s the DR components (Figure 1(a)).^43^ The mechanism by which CAFs contribute to achieving EMT in cancer cells is highly universal across solid tumors. Several studies have revealed that CAFs can produce hepatocyte growth factor (HGF), which further activates the cognate receptor, c-Met, in cancer cells, resulting in a pro-tumorigenic environment by triggering the invasive and metastatic behavior of cancer cells.^44,45^ The contribution of HGF to tumor growth in HNSCC has also been confirmed.^46,47^ There is circumstantial evidence that CAFs are pro-inflammatory due to the activation of nuclear factor kappa B (NF-κB), signal transducer and activator of transcription (STAT) 1 and STAT3 and TGF/SMAD signaling and are engaged in active crosstalk with cancer cells.^48,49^ Moreover, in pancreatic cancer, fibrotic ECM blocks drug infiltration by increasing interstitial fluid pressure and hypovascularization.^50^ Nagathihalli et al. reported that targeted inhibition of STAT3 combined with gemcitabine enhances in vivo drug delivery and therapeutic response, which is characterized by rich DR caused by CAFs and induces treatment resistance. The mechanisms of this increase in tumor drug delivery are not due to depletion of the tumor stroma, but to JAK-STAT3-mediated stromal remodeling and downregulation of Cda in the tumor, which play critical roles in the enhanced delivery of gemcitabine to the tumor, as well as the enhanced activity of gemcitabine within the tumor.^51^ Thus, it may also be possible to improve the efficiency of the drug delivery system, normalize intratumor pressure, and, in some cases, improve the hypoxic environment of the tumor by targeting therapeutic changes in the stromal environment (Figure 2).

## DR and bone invasion of cancers

Abundant DR is prominent at the cancer-bone interface of oral SCC with bone invasion. An et al. reported that Axin2 expression is significantly associated with Snail expression, desmoplasia status, and bone invasion in patients with oral SCC. Moreover, both Axin2 and Snail expression were positively correlated with oral SCC-DR. They concluded that the oncogenic activities of the Axin2-Snail axis are not limited to cancer cells but rather extend to CAFs via regulation of cytokine (CCL5 and IL-8)-mediated cancer-stromal interactions, with further implications for bone invasion and poor prognosis in oral SCC.^52^ On the other hand, an identifiable fibrous stroma with features of bone invasion is less frequently found in benign odontogenic epithelial tumors, such as ameloblastoma, with features of bone invasion.^5^ In the jaw, oral SCC can often invade the bone marrow. Crosstalk with bone marrow cells begins once cancer cells infiltrate the medullary cavity. Regarding cancer-bone marrow progenitor cell interaction, Iwamoto et al.^53^ investigated the transformation of bone marrow-derived macrophages into CAFs in PDAC, promoting tumor progression. Once the bone marrow is invaded, it is conceivable that macrophages, neutrophils, and T cells derived from bone marrow progenitors come into contact with the tumor invasion front, and these cells begin to be involved simultaneously. Future in vivo studies are needed to investigate the TME during bone invasion, focusing on DR formation, its role in bone destruction, and its modulation of osteoclast/osteoblast crosstalk, as these may influence inflammation of the invasion front and the formation of DR in the tumor tissue infiltrating the bone. Moreover, targeting DR at the cancer-bone interface may be a groundbreaking development in the treatment of bone invasion HNSCC cases and potentially other bone-invasive cancers.

## Chemotherapy-induced DR in cancers

It has been known that anticancer drugs induce a DR in the TME. Sun et al. reported that treatment-associated DNA damage responses in benign cells comprising the TME promoted antitumor therapy resistance and subsequent tumor progression in prostate epithelial cancer. They emphasized in vivo evidence of treatment-induced alterations in the tumor stroma, including the expression of a diverse spectrum of secreted cytokines and growth factors. Among these, they investigated that WNT16B is activated in fibroblasts around the tumor through the NF-κB pathway and promotes EMT in neoplastic prostate epithelium through paracrine signaling. Furthermore, WNT16B acts in a cell-nonautonomous manner and promotes the survival of cancer cells after cytotoxic therapy.^54^ Moreover, chemotherapy has been shown to enrich tumor cells in patients with mesenchymal and/or cancer stem cell (CSC) features in various types of cancers. CSCs are intrinsically more resistant to anti-tumor therapy and consequently increase disproportionately following systemic chemotherapy, which is thought to contribute to tumor relapse and treatment resistance.^55^ For example, breast cancers after neoadjuvant chemotherapy are enriched from CD44+CD24− CSCs that also express mesenchymal markers.^56,57^ Additionally, cytotoxic therapies can enrich functionally perturbed CAFs, which are endowed with additional capabilities to enhance cancer stemness, leading to treatment resistance and tumor aggressiveness.^58^ Studies have investigated stemness in the TME after chemotherapy; CAFs are enriched in chemotherapy-treated tumor tissues, wherein they promote cancer growth, treatment resistance, and the self-renewal of CSCs by secreting paracrine factors.^54,59^ Upon stimulation by cytotoxic stress, such as chemotherapy, CAFs can be further induced to secrete pro-stemness cytokines or acquire a senescence-like secretory phenotype. This produces large amounts of pro-stemness chemokines to further enhance tumor stemness and aggressiveness following therapy, and chemotherapy-modulated CAFs secrete a panel of chemokine (C-X-C motif) ligand (CXCL) chemokines to expand CSCs in the treated tumor, leading to paradoxical tumor aggression and treatment failure.^59,60^

## Therapeutic approaches to DR

### Targeting the ECM for enhanced therapeutic penetration

**Figure 3.**
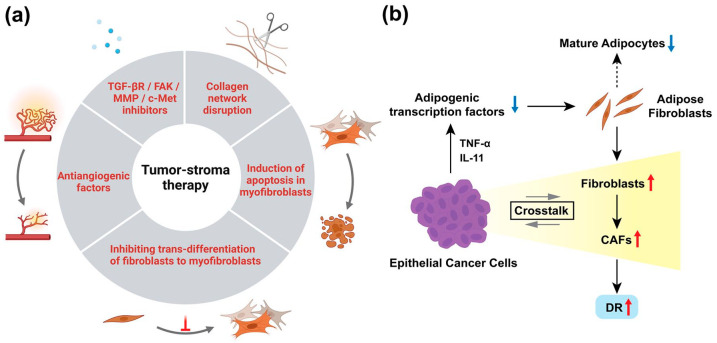
(a) Potential treatment approach targeting tumor stroma. These have not yet been put into practical use, and all are still in the preclinical research phase. (b) One of the strategies of cancer for DR formation. This targets adipogenic transcription factors first via the production of TNF-α and IL-11 by epithelial cancer cells. Then, adipose fibroblasts transit to CAFs instead of the mature fibroblasts, which work on DR formation. DR, desmoplastic reaction; TNF-α, tumor necrosis factor-alpha.

Therapeutic strategies for cancer should be targeted at preventing interactions between cancer cells and the stroma. Potential treatments targeting the stroma include inhibiting the trans-differentiation of fibroblasts to myofibroblasts, inducing apoptosis in myofibroblasts, antiangiogenic factors, and kinds of inhibitors (Figure 3(a)).^61–64^ Netti et al.^65^ investigated an extended collagen network of the ECM in more penetration-resistant tumors and identified collagen as a potential treatment target to improve anti-tumor agent penetration. Moreover, Meng et al. reported that DR surrounding tumors primarily occurs via the action of cytokines (mainly tumor necrosis factor (TNF)-alpha and IL-11) secreted by malignant epithelial cells to inhibit the differentiation of adipose fibroblasts into mature adipocytes. This tumor-induced inhibition of adipocyte differentiation is mediated by the selective inhibition of the expression of essential adipogenic transcription factors.^66^ This tumor milieu can then provide enough room for fibroblasts to grow, which initiates crosstalk with epithelial cancer cells and transition to CAFs, which then work for DR formation (Figure 3(b)).

### Contribution of CAFs and DR for ICI resistance

The CAF secretome is a significant contributor to the ECM and a plethora of cytokines and growth factors that induce immune suppression and form a physical and chemical barrier that induces a T cell-excluding so-called “cold TME.”^67^ CAFs help foster an immunosuppressive TME by promoting regulatory T cells,^39^ which means that the antitumor effect of ICI treatment might be decreased due to the reduced mobilization of T cells into the TME. Targeting the mechanism for CAF-mediated ICI resistance, recent research also investigated the industrial approach. Xu et al. found that salvianolic acid B-loaded PEGylated liposomes can interfere with the activation of CAFs, which play a critical role in the limited therapeutic nanoparticle penetration and suppressive immune TME, by inhibiting the secretion of TGF-β1. After inhibiting the activation of CAFs, collagen deposition in tumors was reduced, and the penetration of nanoparticles in tumors was enhanced. This then induced high expression of CXCL9 and CXCL10 in the TME, which could recruit CD4+, CD8+ T cells, and M1 macrophage and contributed to favorable results via interfering with TGF-β1/Smad signaling to sensitize chemo- and immunotherapy in desmoplastic tumor.^68^

The following provides an overview of evidence concerning inhibitors that target CAFs, significant contributors to DR formation, and their potential role in tumor treatment.

### TGF-β receptor inhibitor

**Figure 4.**
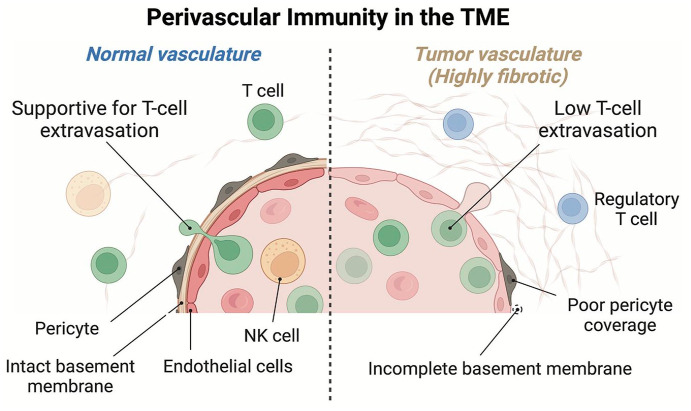
The perivascular immune microenvironment in the TME. The tumor-involved vasculature is frequently immature and often shows difficulty in T cell extravasation and perivascular localization. Moreover, CAF-induced regulatory T cells induce immune exclusion and T cell suppression. CAF, cancer-associated fibroblast; TME, tumor microenvironment.

In a clinical trial, Li et al. reported that a TGF-β receptor (TGF-βR) inhibitor enhanced tumor-infiltrating T cells, and significantly sensitized metastatic CRC to KN046, a drug that blocks PD-L1 and cytotoxic T-lymphocyte-associated protein 4, by targeting the formation of CAFs. Combination therapy using a TGF-βR inhibitor to target CAFs has shown promise in enhancing the efficacy of immunotherapy.^69^ Moreover, Dominguez et al. identified a population of CAFs programmed by TGF-β that express leucine-rich repeat containing 15 proteins, which is valuable for detecting poor responses to ICI treatment in multiple cancer types. Their study has important implications for targeting non-immune elements of the TME to enhance ICI treatment responses.^70^ DR is also characterized by poorly functioning vasculature with variable blood flow through leaky immature vessels, resulting in increased interstitial fluid pressure.^71,72^ Ueno et al.^73^ reported that immature DR in CRC was associated with less angiogenesis and functioned as a barrier to immune cell infiltration to the tumor tissue. Xiao et al.^17^ investigated the mechanisms of ICI resistance, including overcoming the stroma-dependent restriction of T cell extravasation and/or perivascular invasion, reversing immune exclusion, relieving T cell suppression, and altering the immune landscape by reducing myeloid cell accumulation and increasing endogenous CD8+ T and natural killer cell infiltration (Figure 4).

In the head and neck region, Alame et al.^10^ uncovered that the transcriptomic and proteomic data strongly highlighted Notch, TGF-β, and interferon signaling for all salivary duct carcinoma cases in the study, confirming an overall strong DR by measuring α-smooth muscle actin (α-SMA) abundance, the level of which was associated with recurrence-free survival of the patients. Evidence suggests that overexpression of TGF-β leads to remodeling and the replacement of normal glandular parenchyma with interstitial fibrous tissue in a mouse model,^74^ the TGF-β signaling control can be a central game-changer to overcome DR in the TME.

In the clinical trial, NCT04429542 is a phase I/Ib study evaluating the safety, tolerability, and preliminary efficacy of the novel therapeutic agent BCA101, which targets both EGFR and TGF-β, in patients with recurrence/metastatic HNSCC. The trial evaluates both BCA101 monotherapy and its combination with pembrolizumab. The interim report indicates that the objective response rate was 57% in HPV-negative patients and 18% in HPV-positive patients. The disease control rate, including stable disease, was 79% overall (HPV-negative: 89%, HPV-positive: 55%). Grade 3 or higher adverse events occurred in 40% of patients, with skin toxicity being the most common, leading to dose modifications or treatment discontinuation in some cases.^75^ These preliminary data suggest that BCA101 demonstrates promising potential in specific patient populations while underscoring the need for a detailed evaluation of its safety profile.

### Focal adhesion kinase inhibitor

Regarding the negative aspect of DR on the response to ICI treatment, critical obstacles to immunotherapy in PDAC tumors also include a high number of tumor-associated immunosuppressive cells and a unique DR stroma that acts as a barrier to T cell infiltration. Jiang et al. identified hyperactivated focal adhesion kinase (FAK) in neoplastic PDAC cells as a key regulator of fibrotic and immunosuppressive TME. They found that FAK activity was elevated in human PDAC tissues, correlating with high levels of fibrosis and poor CD8+ cytotoxic T-cell infiltration into the TME. Briefly, the FAK inhibitor limited tumor progression, resulting in a doubling of survival in a KPC mouse model (*Kras* and *p53* knockout). This delay in tumor progression was associated with markedly reduced tumor fibrosis and a decrease in the number of tumor-infiltrating immunosuppressive cells. They also found that FAK inhibition caused previously unresponsive KPC mouse models to respond to T-cell-targeted immunotherapy and PD-1 antagonists, indicating that the TME can overcome the DR barrier for T-cell infiltration and the subsequent success of ICI treatment.^76^ This was further supported by a hepatocellular carcinoma (HCC) mouse model: FAK is highly expressed in human HCC and is associated with poor OS and progress-free survival (PFS) in HCC patients and showed the combination of a FAK inhibitor VS4718 and an anti-PD1 antibody had a better effect than monotherapy against HCC.^77^

In HNSCC, Pifer et al. found that the FAK inhibitor increased sensitivity to radiation, enhanced DNA damage, and suppressed homologous recombination and nonhomologous end joining repair in the TP53 mutant, but not wild-type, HPV-negative HNSCC cell lines. The mutant TP53 cisplatin-resistant cell line showed increased FAK phosphorylation compared with the wild-type, and FAK inhibition partially reversed cisplatin resistance.^78^ Although we did not find evidence confirming whether FAK inhibitors can sensitize tumors to ICI therapies, future research should explore the potential of combining these strategies.

In other cancers, the combination of FAK inhibitors and ICIs represents a promising therapeutic strategy to improve antitumor efficacy by modulating the tumor immune microenvironment. Preclinical studies have shown that FAK inhibitors enhance T-cell infiltration and reduce immunosuppression in cancers. A notable clinical trial, NCT02758587, is evaluating the combination of defactinib and pembrolizumab across various cancers, including pancreatic cancer, non-small cell lung cancer (NSCLC), and mesothelioma. The trial investigates biomarkers and mechanisms of action through paired biopsies, aiming to validate the synergistic potential of this combination in ICI-resistant cancers [ClinicalTrials.gov identifier: NCT02758587].

### Matrix metalloprotease inhibitor

Matrix metalloproteases (MMPs) have long been associated with cancer initiation, tumor growth, and metastasis, and have been considered therapeutic targets for the treatment of DR-rich PDAC. A previous study found that combination therapy with marimastat, a soluble broad-spectrum MMP inhibitor, and gemcitabine showed no significant difference compared to gemcitabine alone in median survival time (165.5 vs 164 days) or 1-year survival rates (18% vs 17%). However, an analysis of patients with disease confined to the pancreas (stage I/II) had a median survival of 451 days for those treated with the combination therapy compared to 266 days for those treated with gemcitabine alone. Notably, a small percentage of patients treated with this agent experienced musculoskeletal syndrome.^79^

Although preclinical studies have demonstrated encouraging results, clinical trials of specific MMP inhibitors have shown limited efficacy. This limitation can be attributed to several factors: (1) Mouse model limitations: Differences in MMP biology between humans and mice (e.g., the absence of MMP-1 in mice) hinder preclinical predictions. (2) Dosing challenges: Dosages were based on mitigating musculoskeletal side effects rather than ensuring effective tumor penetration or enzymatic inhibition. (3) Poor understanding of tumor-specific MMP profiles: Variability in MMP expression across tumor types and insufficient recognition of their tumor-promoting roles led to suboptimal trial designs.^80^ In summary, it is unlikely that MMP inhibitors will be developed as standalone treatments. Instead, they are expected to play a supportive role in combination with chemotherapy or immunotherapy to improve overall therapeutic outcomes.

### c-Met inhibitor

The HGF/MET pathway has been identified as a critical role in tumor-stroma interactions. Yasui et al.^81^ demonstrated that stromal cells in PDAC reduce the efficacy of adenoviral therapy via the HGF/MET pathway and that controlling this pathway enhances the effectiveness of adenoviral gene therapy in PDAC. In HNSCC, c-Met inhibition demonstrated significant efficacy by reducing CAF-induced tumor growth, yielding promising results.^46,82^ Although the precise mechanisms of c-Met’s role in HNSCC-DR formation remain unclear, these findings, including the inhibition of CAF function, suggest a potentially critical link between c-Met and DR, particularly through its effects on CAF function.

Although studies specifically targeting c-MET inhibition in HNSCC are limited, preclinical research suggests that the c-MET/HGF pathway could provide therapeutic benefits by modulating tumor progression and metastasis.^83^ Two clinical trials have evaluated MET inhibitors in combination with ICIs, but both primarily focus on NSCLC rather than HNSCC (NCT04139317, NCT03911193). This focus reflects the prevalence of MET exon 14 skipping mutations and MET overexpression in NSCLC, making it a more promising target for those therapies.^84^

### HGF pathway inhibitor

Ficlatuzumab, an anti-HGF antibody, has been investigated in clinical trials for recurrent or metastatic HNSCC, particularly in patients resistant to standard therapies such as cetuximab, platinum-based chemotherapy, and anti-PD-1 inhibitors. A notable phase II trial evaluating ficlatuzumab alone or in combination with cetuximab revealed the efficacy of ficlatuzumab monotherapy, leading to its early discontinuation in this patient population. However, the combination of ficlatuzumab and cetuximab demonstrated promising outcomes, with a median PFS of 3.7 months compared to historical controls. The combination therapy also achieved a higher objective response rate (19%) compared to ficlatuzumab alone (4%). Importantly, patients who were HPV-negative and c-Met-positive showed the most favorable responses, highlighting these subgroups as potential beneficiaries of this combination treatment. Common side effects included hypoalbuminemia, edema, and rash, while serious adverse events such as thromboembolic events and pneumonitis were also observed. These findings suggest that dual targeting of the HGF/c-Met pathway with ficlatuzumab and cetuximab could potentially overcome cetuximab resistance. Further investigation in phase III trials is warranted, particularly in patients with HPV-negative HNSCC.^85^

### NOX1/4 inhibitor

NOX1/4 inhibitors target NADPH oxidase (NOX) 1 and 4, enzymes involved in generating reactive oxygen species, which play critical roles in processes like oxidative stress reduction, fibrosis, tumor growth, and inflammation.^86^ Recent findings presented at ESMO 2024 highlighted the therapeutic potential of setanaxib, a NOX1/4 inhibitor, when combined with pembrolizumab, showing promising results in improving response rates in recurrent or metastatic HNSCC.^87^

Mechanistically, Ford et al.^88^ reported that siRNA-mediated knockdown or pharmacological inhibition of NOX4 with setanaxib normalized CAFs to a quiescent phenotype, facilitating intratumoral CD8+ T-cell infiltration. Unlike TGF-β1, which prevents CAF differentiation but cannot reverse it, NOX4 inhibition uniquely restores CAF function, enhancing immunotherapy sensitivity in CAF-rich tumors.

These findings suggest that NOX inhibition mitigates CAF-mediated resistance to immunotherapy, particularly in tumors with high CAF density, and holds the potential to improve immunotherapy efficacy by modulating the TME to support immune activity.

## Discussion

The influence of the stromal elements in the TME on tumor progression has been of major interest to many oncologists and researchers. In HNSCC, evaluating tumor stroma is important for understanding tumor status, planning adjuvant therapy, and predicting survival. However, a gap remains between molecular research, which has highlighted the clinical significance of tumor stroma, and daily pathology practice, where stromal characteristics are not yet routinely evaluated. Currently, histopathological grading according to the WHO classification, which is based on tumor differentiation or size with or without metastasis, is routinely reported in daily pathology practice. Bridging this gap requires future clinical studies to explore methods like biopsy specimens and/or preoperative imaging to assess stromal characteristics, including DR, and incorporate these findings into recurrence and metastasis risk assessment. Moreover, the development of simple and clear diagnostic tools for DR and the construction of artificial intelligence systems that are useful for observing stromal status will be the next issues to be addressed. It is expected that these tools will not only standardize the diagnostic process but also enable early interventions to increasingly complex diagnostic procedures.

Additionally, the association between the oral microbiota and HNSCC development/progression warrants further investigation.^89^ Microbiological factors may contribute to CAF-mediated DR development and impact prognosis. Advanced techniques like scRNA-seq can elucidate interactions between cell-associated bacteria, host tissue, and immune cells, shedding light on transcriptional pathways linked to inflammation, metastasis, cell dormancy, and DNA repair.^90^

This review highlights DR as a barrier to various antitumor treatments, including ICIs. These investigations provide a strong rationale for combining tumor stroma- and tumor cell-targeted therapies, particularly when treated with ICIs. On the other hand, there is also an opposite interpretation. The conflicting results of several stromal targeting studies show the unfavorable aspects of stromal co-targeting, where the elimination of stromal barriers that influence the delivery of antitumor agents can also potentially drive tumor progression.^50^ Moreover, the above-mentioned evidence also provides a strong impression of the importance of the stromal status in the TME in subsequent treatment decisions for any type of solid cancer.

A limitation of this review is the paucity of research on DR in HNSCC compared to stroma-rich cancers like PDAC. This may be attributed to the relatively smaller number of HNSCC cases and the complexity of factors influencing this disease. However, these pieces of evidence surrounding DR suggest a strong potential for future research in HNSCC. Therefore, this review includes an extensive implication to the latest DR research also conducted in other organs. DR itself is a structure that is secondarily formed under the influence of cancer, and we believe that the formation and role of DR in HNSCC may share several similarities with those in other organs. Integrating insights from DR studies in other cancers could help establish novel diagnostic and therapeutic approaches, ultimately improving outcomes for patients with HNSCC.

## Conclusion

There is a significant gap between molecular research highlighting the clinical relevance of tumor stroma and routine pathology practice, which seldom incorporates stromal evaluation. Evidence suggests that focusing solely on tumor tissue may overlook key tumor-stroma interactions critical to tumor behaviors. Evaluating DR within the TME could provide deeper insights into the progression, recurrence, and metastatic risks of HNSCC. Integrating stromal assessments, particularly DR, into pathological evaluations could enhance prognostic accuracy. This approach may also help predict responses to ICI therapy and support the development of personalized treatment strategies for patients with HNSCC.

## Declarations

**Ethics approval and consent to participate:** Not applicable.

**Consent for publication:** All authors have given consent for publication.

**Availability of data and materials:** Not applicable.
